# *Propionibacterium acnes* biofilm is present in intervertebral discs of patients undergoing microdiscectomy

**DOI:** 10.1371/journal.pone.0174518

**Published:** 2017-04-03

**Authors:** Manu N. Capoor, Filip Ruzicka, Jonathan E. Schmitz, Garth A. James, Tana Machackova, Radim Jancalek, Martin Smrcka, Radim Lipina, Fahad S. Ahmed, Todd F. Alamin, Neel Anand, John C. Baird, Nitin Bhatia, Sibel Demir-Deviren, Robert K. Eastlack, Steve Fisher, Steven R. Garfin, Jaspaul S. Gogia, Ziya L. Gokaslan, Calvin C. Kuo, Yu-Po Lee, Konstantinos Mavrommatis, Elleni Michu, Hana Noskova, Assaf Raz, Jiri Sana, A. Nick Shamie, Philip S. Stewart, Jerry L. Stonemetz, Jeffrey C. Wang, Timothy F. Witham, Michael F. Coscia, Christof Birkenmaier, Vincent A. Fischetti, Ondrej Slaby

**Affiliations:** 1 Laboratory of Bacterial Pathogenesis and Immunology, Rockefeller University, New York, New York, United States of America; 2 Department of Molecular Oncology, Central European Institute of Technology (CEITEC), Masaryk University, Brno, Czech Republic; 3 Department of Microbiology, Faculty of Medicine, Masaryk university, St. Anne’s Faculty Hospital, Brno, Czech Republic; 4 Department of Pathology, Microbiology and Immunology, Vanderbilt University School of Medicine, Nashville, Tennessee, United States of America; 5 Center for Biofilm Engineering, Montana State University, Bozeman, Montana, United States of America; 6 Department of Neurosurgery, St. Anne’s University Hospital, Masaryk University, Brno, Czech Republic; 7 Department of Neurosurgery, University Hospital Brno, Masaryk University, Brno, Czech Republic; 8 Department of Neurosurgery, University Hospital Ostrava, Ostrava University, Ostrava, Czech Republic; 9 Department of Orthopedic Surgery, Stanford University Medical Center, Stanford University, Stanford, California, United States of America; 10 Cedars-Sinai Institute for Spinal Disorders, Los Angeles, California, United States of America; 11 Department of Orthopaedic Surgery, University of California Irvine, School of Medicine, Irvine, California, United States of America; 12 Spine Center, UCSF Medical Center, San Francisco, California, United States of America; 13 Scripps Clinic Division of Orthopedic Surgery, San Diego, California, United States of America; 14 Department of Orthopaedic Surgery, University of California San Diego, San Diego, California, United States of America; 15 Department of Orthopedic Surgery, Kaiser Permanente—San Jose Medical Center, San Jose, California, United States of America; 16 Department of Neurosurgery, The Warren Alpert Medical School of Brown University, Rhode Island Hospital, Providence, Rhode Island, United States of America; 17 Department of Orthopedic Surgery, Kaiser Permanente–Oakland Medical Center, Oakland, California, United States of America; 18 Celgene Corporation, Information Knowledge and Utilization, San Francisco, California, United States of America; 19 Department of Orthopaedic Surgery, David Geffen School of Medicine, University of California, Los Angeles, California, United States of America; 20 Department of Anesthesia, The Johns Hopkins Hospital, Baltimore, Maryland, United States of America; 21 Department of Orthopedic Surgery, University Southern California, Los Angeles, California, United States of America; 22 Department of Neurosurgery, The Johns Hopkins Hospital, Baltimore, Maryland, United States of America; 23 Department of Orthopedic Surgery, OrthoIndy Hospital, Indianapolis, Indiana, United States of America; 24 Department of Orthopedics, Physical Medicine & Rehabilitation, University of Munich (LMU), Munich, Germany; Aarhus Universitet, DENMARK

## Abstract

**Background:**

In previous studies, *Propionibacterium acnes* was cultured from intervertebral disc tissue of ~25% of patients undergoing microdiscectomy, suggesting a possible link between chronic bacterial infection and disc degeneration. However, given the prominence of *P*. *acnes* as a skin commensal, such analyses often struggled to exclude the alternate possibility that these organisms represent perioperative microbiologic contamination. This investigation seeks to validate *P*. *acnes* prevalence in resected disc cultures, while providing microscopic evidence of *P*. *acnes* biofilm in the intervertebral discs.

**Methods:**

Specimens from 368 patients undergoing microdiscectomy for disc herniation were divided into several fragments, one being homogenized, subjected to quantitative anaerobic culture, and assessed for bacterial growth, and a second fragment frozen for additional analyses. Colonies were identified by MALDI-TOF mass spectrometry and *P*. *acnes* phylotyping was conducted by multiplex PCR. For a sub-set of specimens, bacteria localization within the disc was assessed by microscopy using confocal laser scanning and FISH.

**Results:**

Bacteria were cultured from 162 discs (44%), including 119 cases (32.3%) with *P*. *acnes*. In 89 cases, *P*. *acnes* was cultured exclusively; in 30 cases, it was isolated in combination with other bacteria (primarily coagulase-negative *Staphylococcus spp*.) Among positive specimens, the median *P*. *acnes* bacterial burden was 350 CFU/g (12 - ~20,000 CFU/g). Thirty-eight *P*. *acnes* isolates were subjected to molecular sub-typing, identifying 4 of 6 defined phylogroups: IA_1_, IB, IC, and II. Eight culture-positive specimens were evaluated by fluorescence microscopy and revealed *P*. *acnes in situ*. Notably, these bacteria demonstrated a biofilm distribution within the disc matrix. *P*. *acnes* bacteria were more prevalent in males than females (39% vs. 23%, p = 0.0013).

**Conclusions:**

This study confirms that *P*. *acnes* is prevalent in herniated disc tissue. Moreover, it provides the first visual evidence of *P*. *acnes* biofilms within such specimens, consistent with infection rather than microbiologic contamination.

## Introduction

Although first more than 15 years ago, the relationship between intervertebral disc degeneration and chronic, primary infection by *Proprionibacterium acnes* remains controversial. In 11 independent studies published between 2001 and 2016, involving 1188 combined patients undergoing discectomy or microdiscectomy, this Gram-positive bacterial species was isolated from ~25% of resected disc specimens [1–11]. However, these studies had significant methodical differences and the majority was statistically underpowered [1–9] Moreover, the use of qualitative microbiologic techniques—i.e. deeming a specimen *positive* or *negative* for bacterial growth without regard to colony counts—could have led to cultures that were falsely positive for *P*. *acnes* or other organisms. This is due to the concern for low-level bacterial contamination from the surgical environment, the clinical laboratory, or the patients’ skin, as *P*. *acnes* is a nearly ubiquitous human commensal that can colonize the follicle and sebaceous unit at high levels [6, 12].

On the other hand, several of the previous studies reported a low prevalence of culture-positivity in resected lumbar discs [5, 6, 10]. In this light, even if *P*. *acnes* were truly present within a disc, the organism’s mode of growth and the culture techniques employed by the laboratory might underestimate bacterial burden. *P*. *acnes* can be slow to grow and often proliferates *in vivo* as aggregated biofilms, which can be challenging to cultivate *in vitro* and require physical biofilm disassembly (e.g. through sonication or homogenization) prior to microbiologic plating [11–13]. As a result, standard specimen preparation and culture techniques could lead to false negative results. Based on these methodological issues, it is not surprising that the prevalence rate of cultured *P*. *acnes* in previous publications ranges from 0 to 44% [11].

In a recent study from the current authors [11], *P*. *acnes* was identified in 40% of 290 patients undergoing microdiscectomy for disc herniation. That study utilized quantitative culture techniques and it represented the largest series of patients investigated to date. Nevertheless, several methodical weaknesses remained to be addressed, and we had not yet provided direct evidence of *P*. *acnes* biofilms within the discs.

Accordingly, in the current study, we sought to validate and extend our previous work with a subsequent (and larger) independent series of patients undergoing microdiscectomy for lumbar herniation. This study characterizes the prevalence of *P*. *acnes* in resected disc tissue via an improved protocol for quantitative bacterial culture, along with molecular phylotyping of *P*. *acnes* isolates. Moreover, we conduct *in situ* fluorescence imaging of a set of specimens to demonstrate the distribution of the bacteria as a biofilm within the disc tissue.

## Methods

### Study design and patient characteristics

Adults scheduled for microdiscectomy for symptomatic lumbar disc herniation between January 2016 and November 2016 at St. Anne’s University Hospital (Brno, Czech Republic) and University Hospital Brno (Brno, Czech Republic) were prospectively screened immediately prior to surgery and consented for potential participation in this study. To summarize, inclusion criteria covered all adults undergoing microdiscectomy. Exclusion criteria were: immunological compromise; traumatic herniations; the presence of an unknown radiographic mass; and inflammatory or rheumatologic disease. The inclusion and exclusion criteria are described in detail in Capoor et. al. 2016 [11]. Out of 386 patients approached, 8 declined to participate in the study and 5 patients were not included due to rheumatoid arthritis or antibiotic treatment for recurrent urine infection in the one month prior to surgery. Since we were not focused on the follow-up of patients, the only reason for drop-out from our study was zero or insufficient amount of the disc tissue available for microbiology culture. This situation occurred in 5 cases. The epidemiological/clinical data collection included: sex, age, intervertebral segment involved, type of herniation, history of previous spinal surgeries, prior epidural steroid injections, pre-operative pain scores (leg pain [months], back pain [months], NRS (Numerical rating scale for pain) back, NRS leg, Oswestry Disability Index), and development of post-operative discitis. Ethics Committee approvals were obtained for our study from the following hospitals: St. Anne's University Hospital (Brno, Czech Republic) where Hospital Ethics Committee approval was granted (Reference 33V/2015) on June 10, 2015; and, University Hospital Brno (Brno, Czech Republic) where Hospital Ethics Committee approval was granted on May 13, 2015. This study was approved by the Institutional Review Boards of the two participating hospitals and written informed consent was obtained from each patient.

### Surgical specimen collection

All disc samples were obtained using standard operating practices. Specimens were aseptically placed into a closed sterile sample cup to minimize the chances of post-resection contamination [11]. These cups were labeled and immediately transported to the Department of Microbiology at St. Anne’s University Hospital (Brno, Czech Republic). Samples were sterilely divided into two fragments (processed within 2–4 hours post-surgery): the first was utilized immediately for quantitative anaerobic culture, while the second was frozen for future microscopy and other analyses. The size of the resected disc specimens ranged from 3x3x5 mm to 10x5x5 mm.

### Microbiologic culture

The disc fragment for culture was weighed, placed into a Micro Bag (Seward) containing 4 ml of Viande-Levure medium, and homogenized with a Stomacher 80 (Seward) under aseptic conditions. 100 μl of the resultant homogenate was inoculated onto Wilkins Chalgren Anaerobic Agar with 7% sheep’s blood and vitamin K (Hi Media Laboratories). An Anaerobic Work Station Concept 400 (Ruskinn Technology) was utilized for culture; inoculated plates were incubated for 14 days at 37°C with an atmosphere of 80% N_2_, 10% CO_2_, and 10% H_2_. The same amount of the homogenate was also cultured aerobically on Columbia Blood Agar (Oxoid) for 7 days at 37°C in order to detect aerobic bacteria. Following incubation, the bacterial colonies were counted and the quantity of each colonial morphotype was expressed as colony forming units (CFU) per gram of tissue using the Miles and Misra method [14].

### MALDI-TOF mass spectrometry

Taxonomic identification of the above colonies was conducted by matrix assisted laser desorption/ionization time-of-flight mass spectrometry (MALDI-TOF MS). This analysis was performed on a common diagnostic platform, the MALDI Biotyper with FlexControl 3.4 software (Bruker Daltonik), according to manufacturer’s instructions. To summarize, single colonies were applied as a thin film onto a section of a MALDI 96-target plate, overlaid with 1 μl of 70% formic acid, and dried at room temperature. Dried bacteria were overlaid with 1 μl of matrix solution, saturated α-cyano-4-hydroxycinnamic acid solution in acetonitrile–water–trifluoroacetic acid (50:47.5:2.5, v/v), and allowed to dry before spectral acquisition. Mass spectra were processed using the BioTyper 3.1 software, and manufacturer-recommended cut-off scores were employed for identification (≥2 indicating species-level identification; 1.7–1.999 indicating genus-level identification, and <1.7 indicating no identification). Colonies with isolates an initial score <1.7 were retested, and the highest score was used for identification.

### Confocal scanning laser microscopy and SYTO9 staining

Ten frozen disc specimens were selected primarily by a sample size > 0.5 g. These included 8 *P*. *acnes*-only specimens (4 from men and 4 from women) with a bacterial load > 10^3^ CFU/g (out of 38 total), as well as 2 negative controls with no culture growth. Frozen fragments were transported on dry ice to the Center for Biofilm Engineering at Montana State University (CDC PHS Permit 2016-05-119) for confocal laser scanning microscopy (CSLM). Disc tissue was defrosted and fixed using 4% paraformaldehyde (Electron Microscopy Science); cryoembedded in Optimal Cutting Temperature medium (Saukura Finetek); cryosectioned at -20°C into 7–10 μm sections; and placed onto Superfrost Plus glass slides. For nucleic acid labeling, slides were stained with the fluorescent DNA maker SYTO9 (ThermoFisher Scientific) and imaged on a Leica SP5 confocal scanning laser microscope, with 3-D reconstruction by Imaris software.

### Fluorescent *in-situ* hybridization

Two fluorescent *in-situ* hybridization (FISH) probes were employed in this study: the general eubacterial 16S rRNA probe EUB338 (GCTGCCTCCCGTAGGAGT) and the *P*. *acnes*-specific 16S rRNA probe PAC16s598 (GCCCCAAGATTACACTTCCG). These oligonucleotides (Sigma-Aldrich) were labeled at both the 5’ and 3’ ends with either CY5 (for EUB338) or CY3 (for PAC16s598). The taxonomic specificity of these probes was first validated through experiments with cultured *P*. *acnes*, *Staphylococcus aureus*, *S*. *epidermidis*, and *Pseudomonas aeruginosa*. For FISH analysis, the resected discs were sectioned onto glass slides (as described above) and re-fixed with 4% paraformaldehyde (20 minutes at room temperature), followed by a deionized water (DIW) rinse. Slides were next treated with 1 mg/mL lysozyme (Acros Organics) and 30 units/mL achromopeptidase (Wako) for 20 minutes at 37°C in a humidified chamber. After another DIW wash, the sections were dehydrated in a graded ethanol series (50%, 80%, 100%; 3 minutes each) and dried with filtered compressed air.

FISH probes (2ng/μL each, final concentration) were added to a hybridization buffer (900μL filter-sterilized H_2_O, 360μL 5M NaCl, 40μL 1M Tris/HCl, 700μL formamide, and 2μL 10% sodium dodecyl sulfate). Prepared slides were stained with the probes overnight at 46°C, followed by 20 minutes at 48°C in wash buffer (700μL 5M NaCl, 1mL 1M Tris/HCl, 50μL of 10% SDS, made up to 50 mL with filter-sterilized H_2_O). Slides were then rinsed with ice-cold water and dried with compressed air. A drop of Prolong Gold anti-fade solution (Life Technologies) was added to the section, which was covered with a coverslip and allowed to dry overnight at room temperature. Sections were visualized with a Nikon Eclipse E800 microscope equipped with a CY3 and CY5 filter cube.

### *P*. *acnes* phylotype analysis

Genomic DNA from *P*. *acnes* isolates was purified using the QIAamp DNA mini kit (Qiagen). Phylotyping was conducted according to the MLST8 scheme and determined by a recently described multiplex PCR assay [15]. Refer to the cited publication for primer information and amplification conditions. A C1000 Touch^TM^ Thermal Cycler (Bio-Rad) was employed for PCR.

### Statistical analysis

Culture positivity, bacterial identity, and quantitative organism burden (CFU/gram) were correlated to the patients’ documented clinical parameters with the use of the Prism 5 software package (GraphPad Software, Inc.). Analyses of categorical variables were performed with a Fisher’s two-sided exact test, and analysis of patient age was performed with a non-parametric Mann-Whitney U test (significance level set at p = 0.05).

## Results

### Bacterial culture analysis

Resected disc specimens from 368 patients with lumbar herniation were subjected to extended anaerobic culture. Bacterial growth was observed and identified by MALDI-TOF for 162 of 368 specimens (44%), with no colonies observed for the other 206. Among positive specimens, *P*. *acnes* was present in 119 cases (32.3%); and, non-P. acnes bacteria was present in 42 cases (11.4%). *S*. *saccharolyticus* comprised 11 (3%), *S*. *epidermidus* comprised 10 (2.7%), and *S*. *haemolyticus* 3 (0.8%) of these 42 cases; and, all the remaining species represented either one or two cases. For specimens with growth of *P*. *acnes*, a majority (89/119, 75%) yielded only this organism, without any additional bacterial species present. Conversely, 30 specimens (34%) demonstrated mixed growth of 2 or more bacterial species. Of these *P*. *acnes* with *S*. *epiderm*idus, *P*. *acnes* with *S*. *haemolyticus*, *P*. *acnes* with *S*. *hominis*, and *P*. *acnes* with *S*. *warneri* each accounted for 4 cases (1.1%). 38 *P*. *acnes* cases (10.3%) were found to be ≥ 1000 CFU/g: 31 where the species was exclusive, 4 in combination with *Staphylococcus* (i.e., 2 with *S*. *warneri*, 1 with *S*. *haemolyticus*, and 1 with *S*. *pasteuri*), and 1 with *P*. *granulosum*. Also, 13 cases of other bacteria (3.5%) were found, in aggregate, to be ≥1000 CFU/g, 5 of which were in combination with *P*. *acnes*. Of the remaining 8 cases (2.2%), 4 cases were *Staphylococcus* only (i.e., 2 were *S*. *epidermidus*, 1 of *S*. *epidermidus* with *S*. *hominis*, and 1 was *S*. *warneri*), 2 were primarily *Streptococcus* (i.e., 1 of *S*. *mitis* and 1 of *S*. *mitis* with *S*. *parasanguinis*. and *A*. *graevenitz*), 1 was *C*. *tuberculostearicum*, and 1 was *Enterococcus gallinarum* (refer to Table 1 for a detailed summary of this information). Aerobic cultivation did not lead to any additional significant findings. Cultivation neither increases the number of positive samples nor extends the spectrum of detected microbes.

**Table 1:** Summary of the microorganisms isolated in 162 cases from 368 patient intervertebral disc specimens by anaerobic culture.

			≥ 1000 CFU/g			
					Aggregate of	
	Positive Cases		*P. acnes*		Other Bacteria	
Isolated microorganism	N	%	N	%	N	%
***Propionibacterium acnes* cases**	**119**	**32.3%**	**38**	**10.3%**	**5**	**1.4%**
***P*. *acnes* only**	**89**	**24.2%**	**31**	**8.4%**		
***P*. *acnes* with *Staphylococcus* only**	**22**	**6.0%**	**5**	**1.4%**	**4**	**1.1%**
*P*. *acnes*, *S*. *aureus*	1	0.3%				
*P*. *acnes*, *S*. *capitis*	2	0.5%				
*P*. *acnes*, *S*. *epidermidus*	4	1.1%	1	0.3%		
*P*. *acnes*, *S*. *haemolyticus*	4	1.1%	2	0.5%	1	0.3%
*P*. *acnes*, *S*. *hominis*	4	1.1%				
*P*. *acnes*, *S*. *pasteuri*	1	0.3%	1	0.3%	1	0.3%
*P*. *acnes*, *S*. (unspecified)	1	0.3%				
*P*. *acnes*, *S*. *warneri*	4	1.1%	1	0.3%	2	0.5%
*P*. *acnes*, *S*. *intermedius*	1	0.3%				
***P*. *acnes* with other**	**8**	**2.2%**	**2**	**0.5%**	**1**	**0.3%**
*P*. *acnes*, *P*. *granulosum*	1	0.3%	1	0.3%	1	0.3%
*P*. *acnes*, *P*. *granulosum*, *F*. *magna*	1	0.3%	1	0.3%		
*P*. *acnes*, *P*. *acidifaciens*, *S*. *hominis*	1	0.3%				
*P*. *acnes*, *S*. *epidermidus*, *S*.*viridans*	1	0.3%				
*P*. *acnes*, *S*. *haemolyticus*, *S*. *oralis*	1	0.3%				
*P*. *acnes*, *S*. *hominis*, *Paenibacillus*	1	0.3%				
*P*. *acnes*, *S*. *mitis*	1	0.3%				
*P*. *acnes*, *S*. *oralis*, *Veilonella dispar*	1	0.3%				
**Non-*P*. *acnes* bacteria cases**	**43**	**11.7%**			**8**	**2.2%**
***Staphylococcus* only**	**32**	**8.7%**			**4**	**1.1%**
*S*. *capitis*	1	0.3%				
*S*. *epidermidus*.	7	1.9%			2	0.5%
*S*. *epidermidus*. *S*. *haemolyticus*	1	0.3%				
*S*. *epidermidus*. *S*. *hominis*	1	0.3%			1	0.3%
*S*. *haemolyticus*	3	0.8%				
*S*. *hominis*	2	0.5%				
*S*. *lugdunensis*	1	0.3%				
*S*. *pasteuri*	2	0.5%				
*S*. *saccharolyticus*	11	3.0%				
*S*. (unspecified)	2	0.5%				
*S*. *warneri*	1	0.3%			1	0.3%
***Staphylococcus* with other**	**3**	**0.8%**				
*S*. *epidermidus*, *A*. *odontolyticus*	1	0.3%				
*S*. *haemolyticus*, *Arthrobacter* (unspecified)	1	0.3%				
*S*. *haemolyticus*, *Rothia amarae*	1	0.3%				
***Streptococcus***	**3**	**0.8%**			**2**	**0.5%**
*S*. *mitis*	1	0.3%			1	0.3%
*S*. *viridans*	1	0.3%				
*S*. *mitis*, *S*. *Parasanguinis*., *A*. *graevenitz*	1	0.3%			1	0.3%
***Corynebacterium***	**2**	**0.5%**			**1**	**0.3%**
*C*. *minutissimum*	1	0.3%				
*C*. *tuberculostearicum*	1	0.3%			1	0.3%
**Other**	**3**	**0.8%**			**1**	**0.3%**
*Agromyces* (unspecified)	1	0.3%				
*Enterococcus gallinarum*	1	0.3%			1	0.3%
*Rothia dentocariosa*	1	0.3%				
**Total**	**162**	**44.0%**	**38**	**10.3%**	**13**	**3.5%**

***Notes*:** for *P. acnes* ≥ 1000 CFU/g applies to that species; for all other bacteria listed, an aggregate of the species found is used. “(unspecified)” refers to a species not identified within the preceding genus.

### Quantitative burden of *P*. *acnes* in disc specimens

**Fig 1:**
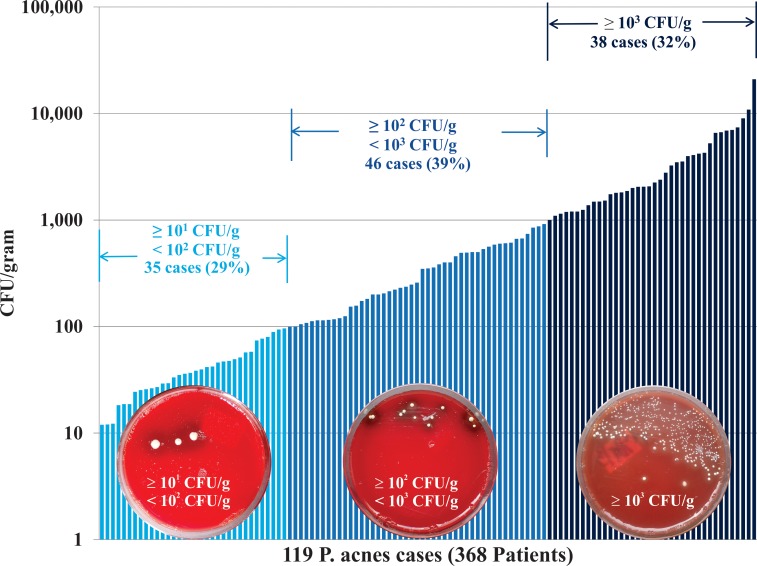
Distribution of *P*. *acnes* colony counts in culture-positive disc tissue specimens.

The observed organism burden from the 119 *P*. *acnes*-positive specimens varied significantly from case to case. Quantitative *P*. *acnes* counts ranged from 12 to 20952 CFU/g, with a median of 350 CFU/gram. We noted 38 cases (32%) with *P*. *acnes* colony counts ≥ 10^3^ CFU/gram; 46 cases (39%) with 10^2^–10^3^ CFU/gram; and the remaining 35 cases (29%) with 10^1^–10^2^ (Fig 1).

### *In situ* determination of bacteria by CSLM

**Fig 2:**
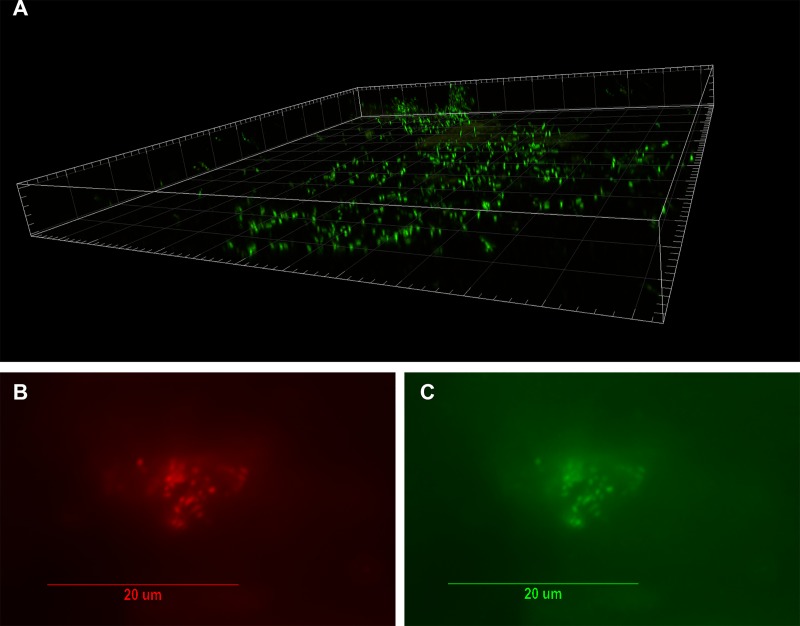
Visualization of bacterial biofilm in the disc tissue by CSLM and confirmation of *P*. *acnes* by FISH. A. Three dimensional reconstructed CSLM image of biofilm bacteria stained with a DNA stain (SYTO9, green) in a disc tissue sample (#4, Table 2). B-C. The presence of *P. acnes* biofilms in this sample verified using FISH. Epifluorescence micrographs of a biofilm cluster showing red fluorescence from the CY5-labeled EUB338 general eubacterial probe (B) and green fluorescence from the CY3-labled *P. acnes*-specific probe (C). Co-localization of the red and green fluorescence indicates that all of the bacteria in this biofilm were *P. acnes*.

Given the high burden of cultured *P*. *acnes* in many specimens, we next sought to investigate the *in situ* relationship of the organisms to the disc tissue. A set of 8 specimens with > 1000 CFU/g (along with 2 culture-negative controls) were selected for visualization, first via confocal scanning laser microscopy (CSLM) and the SYTO9 DNA dye. Bacterial biofilms were detected in 7 out of 8 culture-positive specimens tested (Table 2). A three-dimensional reconstruction of a typical biofilm is provided in Fig 2A (from Sample 4). The bacteria are present within the body of the herniated intervertebral disc, not on the surface (as would be expected for a perioperative contaminant that became associated with the tissue during resection). No bacteria were visualized in the culture-negative control samples.

**Table 2:** Characteristics of the disc samples evaluated by microscopic methods.

			Previous	Sample	SYTO-9	FISH
Sample	CFU/	Sample	Spine	Weight	Biofilm	Biofilm
Number	gram	Description	Surgery	mg	observed	observed
**1**	**0**	**Control**	**No**	**950**	**No**	**No**
**2**	**0**	**Control**	**No**	**1070**	**No**	**No**
3	1097	*P*. *acnes* only	No	2480	Yes	No
4	9016	*P*. *acnes* only	No	1930	Yes	Yes
5	3977	*P*. *acnes* only	No	1770	Yes	Yes
6	1378	*P*. *acnes* only	No	900	Yes	Yes
7	2069	*P*. *acnes* only	No	580	Yes	Yes
8	1193	*P*. *acnes* only	No	570	Yes	Yes
9	3482	*P*. *acnes* only	No	1700	No *	Yes
10	4073	*P*. *acnes* only	No	550	Yes	No

* Individual bacteria were observed.

### *In situ* identification of *P*. *acnes* by FISH

**Fig 3:**
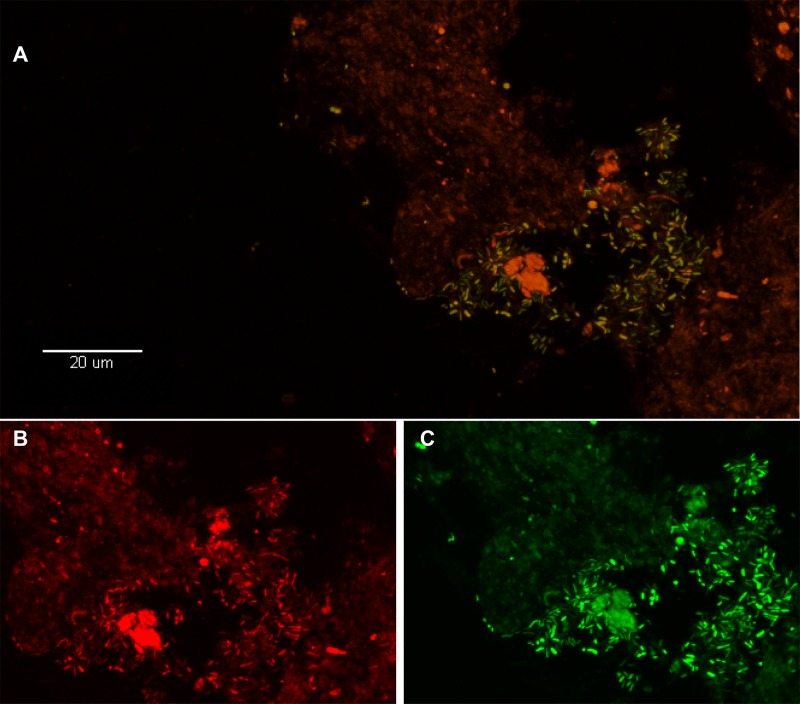
Visualization of *P*. *acnes* biofilm in the disc tissue by use of FISH. A. This color-combined image shows the “pocket” of green fluorescent *P. acnes* cells (biofilm) near the center right of the image (disc tissue sample #8, Table 2). The presence of *P. acnes* biofilms in this sample was verified using FISH. B-C. Red fluorescence is the general eubacterial probe (B) and green is the *P. acnes* probe (C). The B/C image is a zoom of A showing fluorescence from the red and green channels separately. Almost all of the cells in A are emitting both red and green fluorescence indicating that they are *P. acnes*.

To complement the SYTO9 images, further fluorescence microscopy was conducted with 16S rRNA FISH probes targeting either a ubiquitous eubacterial sequence (EUB338) or *P*. *acnes* specifically (PAC16s598). These probes were applied to all 8 of the disc specimens described above, for which > 1000 CFU/g of *P*. *acnes* were cultured and biofilms were visualized by SYTO9 staining and CSLM with the exception of Sample 9. The presence of bacterial biofilms was confirmed in 6 of the 8 specimens with the EUB338 probe, which also stained positively with PAC16s598. Two examples of images (with both probes) are depicted in Fig 2 and Fig 3. Overall, these data further demonstrate the presence of *P*. *acnes* biofilm-aggregates in resected disc specimens with corresponding culture positivity.

### Phylotyping of *P*. *acnes* isolates from intervertebral discs

We phylotyped 38 isolates of *P*. *acnes* from discs with >1000 CFU/g to gauge whether any clade-specific associations exist. Type IA_1_ *P*. *acnes* was identified in 15 (39%) cases, type IB in 9 (24%) cases, type IC in 1 (3%) case, and type II in 13 (34%) cases. No dominant phylotype was identified in either the total group of 38 strains or the subset with visualized biofilms in resected discs.

### Patient characteristics and *Propionibacterium acnes* positivity

A total of 368 patients were enrolled (222 males and 146 females) in the study, at an average age of 49.3 ± 13.6 years and an age range extending from 20 to 83. Between the two surgical centers, 245 patients were enrolled at University Hospital Brno and 123 at St. Anne’s University Hospital; there were no significant differences between the two centers with regards patient age, sex, or disease-specific clinical data. A medical history of previous spinal surgery was noted in 58 (16%) of cases, though none of the patients developed clinically evident post-operative discitis after these surgeries.

To provide clinical context to the microbiologic culture results, we sought to correlate *P*. *acnes* positivity to the patients’ clinical parameters. *P*. *acnes* positive patients were significantly younger than *P*. *acnes* negative patients (46.7 versus 50.5 years; p = 0.0380). Roughly 39% of 222 males were *P*. *acnes* positive compared to 23% of 146 females (p = 0.0013). There were no significant differences between *P*. *acnes* positive and negative patients in prevalence of previous spinal surgery, distribution of herniation type, affected intervertebral level, or pre-surgical pain scores. Results are summarized in Table 3.

**Table 3:** Patient and clinical characteristics in relation to *P*. *acnes* positivity.

					*P*. *acnes*				*P*. *acnes*				
					Positive				Negative				
Parameters	N		%		N		%		N		%		p-value
**Number of patients**	368		100%		119		32%		249		68%		
Male	222		60%		86		39%		136		61%		**0.0012**
Female	146		40%		33		23%		113		77%		
**Age**													
Median ± SD	49.3	±	13.6	y	46.7	±	12.4	y	50.5	±	13.7	y	**0.0145**
**Previous disc surgery**													
Yes	58		16%		20		34%		38		66%		
No	310		84%		99		32%		211		68%		
**Type of herniation**													
Protrusion	55		15%		15		27%		40		73%		
Extrusion	181		49%		57		31%		124		69%		
Sequestration	132		36%		47		36%		85		64%		
**Intervertebral level**													
Multiple level procedures^A^	17		5%		7		41%		10		59%		
L1/L2	1		0%		0		0%		1		100%		
L2/L3	9		2%		0		0%		9		100%		
L3/L4	28		8%		6		21%		22		79%		
L4/L5	149		40%		55		37%		94		63%		
L5/L6	5		1%		2		40%		3		60%		
L5/S1	159		43%		49		31%		110		69%		
**Presurgical Pain Scores**													
** (All patients)**													
***Back Pain***													
Number of patients	298		81%		106		36%		192		64%		
Duration, Months ± SD	16.2	±	41.1	m	15.8	±	35.2	m	16.5	±	44.2	m	
***Leg Pain***													
Number of patients	67		18%		15		22%		52		78%		
Duration, Months ± SD	6.1	±	8.9	m	7.5	±	14.8	m	5.6	±	6.5	m	
***Φ NRS Back***													
Number of patients	79		21%		18		23%		61		77%		
Φ NRS Score, Median ± SD	4.8	±	2.1		5.4	±	2.3		4.6	±	2.1		
***Φ NRS Leg***													
Number of patients	71		19%		17		24%		54		76%		
Φ NRS Score, Median ± SD	6.1	±	1.8		6.4	±	2.2		6.0	±	1.7		
***Oswestry Disability Index***													
Number of patients	85		23%		21		25%		64		75%		
Oswestry Score, Median ± SD	40.3	±	17.7		45.4	±	18.2		42.9	±	17.7		

^A^ These procedures are excluded in the disc levels that follow.

## Discussion

Over the last 15 years, it has been hypothesized that intervertebral disc degeneration and the accompanying chronic lower back pain may be associated—at least in some cases—with chronic subclinical bacterial infection by *P*. *acnes* [16]. A recently described animal model demonstrates the ability of *P*. *acnes* to induce disc degeneration [17, 18]. However, in microbiologic culture data, contamination is difficult to exclude entirely, as *P*. *acnes* is a common skin commensal. The current study seeks to resolve this dilemma through an improved protocol for quantitative bacterial culture, *in situ* imaging of resected disc tissue, and molecular phylotyping of *P*. *acnes* isolates.

In our previous study [6], we were first to utilize quantitative bacterial culture for detection of *P*. *acnes* in disc samples. Here we addressed several weaknesses: (i) weighing the disc tissue prior to homogenization to allow for a precise CFU/gram determination, rather than CFU/ml homogenate; (ii) utilizing a Stomacher 80 for disc tissue homogenization, rather than a manual mortar and pestle; (iii) increasing the plating volume from 10 μl (using a loop) to 100 μl (using a pipette); and (iv) confirmation of bacterial identity by MALDI-TOF MS, rather than biochemical tests. These improvements can help facilitate a higher level of standardization in the future, providing investigators with means for inter-study reproducibility and comparability. In particular, the inclusion of a biofilm disassembly step—such as tissue homogenization through the use of a Stomacher—is a component we believe is crucial for cultures to accurately reflect tissue burden and avoid false negatives.

With this improved culture protocol, we sought to confirm the prevalence of cultured *P*. *acnes* observed in our previous study [6], this time with an even larger population of patients undergoing microdiscectomy for lumbar disc herniation. In comparison to this earlier work, we observed a slightly lower prevalence rate of *P*. *acnes* (32% vs. 40%), which may be a result of the improved methodology and mainly utilizing a Stomacher 80 that is less vulnerable to introduce contamination than mortar and pestle, which was used for disc tissue homogenization in our previous study. Overall, both studies are in agreement with a majority of previous publications that describe *P*. *acnes* as the species most frequently isolated from lumbar disc tissue by anaerobic culture, including Stirling et al. (44%) [9], Arndt et al. (35%) [4], and Albert et al. (40%).

The observed colony-counts in the 119 *P*. *acnes* positive specimens ranged significantly, from 12 to 20952 CFU/gram (median = 350 CFU/gram). Although one might expect that *true* infections are more likely associated with higher colony-counts, we sought to provide direct visual confirmation of *P*. *acnes* biofilm. We selected 10^3^ CFU/g as a positivity threshold to attempt fluorescence microscopy, although samples with lower bacterial counts could certainly include true positives, as well (Table 2). To our knowledge, this study represents the first three-dimensional reconstruction of an intervertebral disc biofilm by use of CSLM, as well as direct confirmation of *P*. *acnes* biofilm by organism-specific FISH (Fig 2 and Fig 3) [19].

Even at a tissue burden of 10^3^ CFU/g, identifying organisms in histologic section represents a distinct challenge, as they can readily evade visual detection at this level. Our ability to observe biofilms in 7/8 specimens suggests that the actual bacterial burden may exceed the measured CFU/g values, even with the biofilm disassembly step. It is important to note that, unless a homogenization step disperses a biofilm entirely to individual organisms (highly unlikely), a CFU/g measurement will be less than the true organism/gram value. In fact, colony counts could further underestimate the *in situ* bacterial burden if the biofilm contains a mix of viable and nonviable organisms (which histologic observations cannot assess). In either case, our findings highlight the need for laboratory procedures that take into account the biofilm mode of growth. Because disc specimens were cryoembedded as intact fragments, one would expect contamination to be limited to the surfaces of the specimens. Instead, bacteria were observed in the interior of the frozen blocks. This distribution of *P*. *acnes* within the body of the intervertebral disc fragment provides strong evidence against contamination from the skin or external environment. Given that FISH sensitivity is lower than DNA staining with SYTO, it is not unexpected that with respect to case 3 and 10 we did not confirm the biofilm observed with DNA stain by FISH.

We further applied the MLST8 phylotyping scheme that distinguishes between *P*. *acnes* strains classically associated with inflammation/acne and those associated with benign—or perhaps even beneficial—skin colonization [20]. Type IA_1_ is commonly found in abundance in inflamed skin, whereas Type II is generally associated with blood, endodontic infections, and normal skin [20]. Focusing on strains cultured in high density (>1000 CFU/g), we observed a distribution of phylotypes rather than a single predominance. The results in this study are similar to those of Rolasson et. al. [21], who (through single-locus *recA* analysis) observed that 50% of disc isolates belonged to phylotype II (34% here) and 42% to phylotype IA_1_ (39% here) [9]. By contrast, whereas our study detected 24% phylotype IB, Rolasson et. al. observed only 9%. Together, these studies suggest that no specific *P*. *acnes* phylotype is more prone to disc infection, although continued characterization of disc isolates will provide a more detailed phylotype distribution.

Finally, we evaluated potential associations between *P*. *acnes* culture positivity and the clinical parameters of studied patients. In agreement with our previous study [6], patients with *P*. *acnes* culture positivity were younger than culture-negative patients. One explanation for these findings is that *P*. *acnes* might accelerate age-related disc degenerative and associated symptoms. Furthermore, we observed a significantly higher prevalence of *P*. *acnes* in disc specimens from males, a finding whose significance remains to be elucidated. From a clinical utility and outcomes perspective, pre-surgical pain scores did not discriminate between *P*. *acnes* positive and *P*. *acnes* negative patients. However, long-term post-surgical patient reported outcomes scores may provide further insight in this regard.

Overall, one of the most significant implications of our findings is the way they challenge traditional paradigms of *infection* (typically inflammatory and pathological) versus *colonization* (non-inflammatory and physiological). Intervertebral discs are traditionally viewed as sterile spaces, so the presence of viable bacteria either (1) represents a non-physiologic process or (2) re-defines the anatomic boundaries of the normal human microbiome. The association of these organisms with disc herniation—as either an upstream or downstream event—suggests the former. While the organisms do not appear to induce a leukocytic infiltrate, the critical question facing clinicians remains unresolved: might they otherwise contribute to the pain symptoms of chronic lower back pain?

An additional noteworthy finding of the current study, as well previous publications [1–11], was the isolation of Gram-positive bacteria other than *P*. *acnes*, specifically *Staphylococci* from the resected discs. Although not closely related phylogenetically, *P*. *acnes* and *Staphylococci* share certain functional traits, such as their ability to colonize the skin and the propensity of various species/strains to form biofilms [22]. In our 368 patient study we cultured *P*. *acnes* in 119 cases and *Staphylococci* in 61 total cases. Of the 8 cases of *Staphylococci* with colony counts ≥ 1000 CFU/g no *Staphylococci* species appeared more than twice. On other hand, *P*. *acnes* with colony counts ≥ 1000 CFU/g was isolated in 38 cases. Therefore, we conclude that *P*. *acnes* is the only significant species cultivated from the disc.

The current study has several limitations that could be addressed in future work. Although we performed MLST8 typing of isolates, higher resolution sequencing of additional strains may be needed to determine whether any distinct clades (or other genetic determinants) are associated with disc colonization/infection. *P*. *acnes* populates several cutaneous niches, but can also colonize the upper respiratory mucosa, gastrointestinal tract, conjunctiva, and external ear canal. The organism is likewise associated with device-associated infections, prostate cancer, and sarcoidosis [23]. However, although 119 genomes of *P*. *acnes* are currently available in Genbank [24, 25], a large predominance represent skin isolates. As a result, molecular methods to phylotype *P*. *acnes* strains are potentially biased and could underestimate the diversity of strains from other body sites. In this light, additional sequencing of disc isolates could provide general insight into the population structure of the species.

From a clinical perspective, our study only collected pre-operative pain scores. To evaluate potential clinical utility, studies analyzing both pre- and post-surgical pain scores in relation to *P*. *acnes* counts (CFU/g) have to be conducted. Also, studies on patients who undergo a second disc surgery, due to failure of their first procedure, could support the potential clinical utility of *P*. *acnes* detection. In this subset of patients, it would be insightful to compare quantitative data between the procedures, especially if CFU/g or other molecular values were higher for the second surgery. For the broader population of patients experiencing chronic lower back pain [26, 27] it will be important to determine whether *P*. *acnes* is exclusive to the herniated portion of the disc tissue (*i*.*e*., *in specific locations within a disc*) or distributed throughout the whole disc. A study utilizing disc tissue from spinal fusion surgeries could provide necessary insight.

The preceding paragraph also alludes to an inherent limitation of the research strategy underlying our manuscript, which should be understood in order not to make conclusions that are currently unsupported by our data. We have studied disc material from patients undergoing lumbar disc surgery either because of failed conservative treatment or because of clinically relevant motor deficits. Doing so permitted us to perform a large-scale epidemiological study without requiring volunteers to undergo an investigatory procedure.

Decompressive surgery for herniated nucleus pulposus typically targets radicular symptoms (paralysis, intractable neuropathic pain) and not low back pain, even though low back pain may be a minor component of these patients’ overall complaints. The immediate and short-term success of disc surgery is measured by the alleviation of these radicular symptoms and if a subset of these patients’ progress to become chronic low back pain (CLBP) patients, it is not typically in the immediate follow-up. So in essence, our findings deliver an epidemiological situation report on a selective group of middle-aged adults with specific consequences of Degenerative Disc Disease (DDD) that are not necessarily representative of the population at large and most probably not of the typical CLBP patient or the failed back surgery patient / failed fusion patient.

It would therefore be premature on the basis of our current data to claim that there is a direct link between low-grade disc infections with P. acnes and CLBP or failed lumbar arthrodesis surgery, even though there is an increasing body of literature pointing into that direction [28–30].

P. acnes behaves rather differently from most other bacterial pathogens. In contrast with rapidly progressive pyogenic infections that result in severely septic conditions, P. acnes infections tend to result in slow, low-grade infections [31–34]. The germ has also been tied to disease conditions that carry immunological aspects such as SAPHO syndrome and sarcoidosis [35–42]. P. acnes may in certain situations become an intracellular infectious agent that can survive and even travel within human macrophages [43, 44]. At this time, though the findings are suggestive, we cannot, and do not, recommend all patients undergoing discectomy send disc material for culture as current routine lab culturing may not be sufficient to indicate an infection. Additionally, until trials are done, we do not recommend antibiotics are needed. Further assessments may identify subsets of individuals where these two points could be considered.

In conclusion, this represents the largest study to date demonstrating a high prevalence of *P*. *acnes* in the disc tissue of patients undergoing microdiscectomy. Moreover, it provides the first microscopic evidence of *P*. *acnes* biofilm in the resected lumbar spine disc tissue. It is still possible that, among *P*. *acnes* culture-positive specimens, some originated through perioperative contamination. However, given the specimens with a high organism burden and the microscopic evidence of biofilms, we conclude that at least a sub-set of herniated intervertebral discs truly become populated with *P*. *acnes*.

## Supporting information

S1 File Underlying Data. (XLSX)
